# Continuous Multidisciplinary Care for Patients With Orofacial Clefts—Should the Follow-up Interval Depend on the Cleft Entity?

**DOI:** 10.1177/10556656211035253

**Published:** 2021-08-19

**Authors:** Anna K. Sander, Elisabeth Grau, Anita Kloss-Brandstätter, Rüdiger Zimmerer, Michael Neuhaus, Alexander K. Bartella, Bernd Lethaus

**Affiliations:** 9180 University Hospital Leipzig, Leipzig, Germany

**Keywords:** cleft lip palate, cleft entity, multidisciplinary, follow-up

## Abstract

**Objective:**

The multidisciplinary follow-up of patients with cleft lip with or without palate (CL/P) is organized differently in specialized centers worldwide. The aim of this study was to evaluate the different treatment needs of patients with different manifestations of CL/P and to potentially adapt the frequency and timing of checkup examinations accordingly.

**Design:**

We retrospectively analyzed the data of all patients attending the CL/P consultation hour at a tertiary care center between June 2005 and August 2020 (*n* = 1126). We defined 3 groups of cleft entities: (1) isolated clefts of lip or lip and alveolus (CL/A), (2) isolated clefts of the hard and/or soft palate, and (3) complete clefts of lip, alveolus and palate (CLP). Timing and type of therapy recommendations given by the specialists of different disciplines were analyzed for statistical differences.

**Results:**

Patients with CLP made up the largest group (*n* = 537), followed by patients with cleft of the soft palate (*n* = 371) and CL ± A (*n* = 218). There were significant differences between the groups with regard to type and frequency of treatment recommendations. A therapy was recommended in a high proportion of examinations in all groups at all ages.

**Conclusion:**

Although there are differences between cleft entities, the treatment need of patients with orofacial clefts is generally high during the growth period. Patients with CL/A showed a similarly high treatment demand and should be monitored closely. A close follow-up for patients with diagnosis of CL/P is crucial and measures should be taken to increase participation in follow-up appointments.

## Introduction

The complete rehabilitation of patients with orofacial clefts generally requires a high therapeutic effort by different specialties. Areas of concern comprise the multistep surgical reconstruction, nasal airflow, hearing and pathology of the middle ear, speech and pronunciation, feeding, development of the jaws, dental malposition, and esthetic appearance (Godbersen, 1997; Taib et al., 2015; Padovano et al., 2020). The surgical reconstruction of the lip, the nostril, the oral vestibule, the nasal floor, and the hard and soft palate usually takes place within the first year of life. During this period, there is an intensive supervision by the maxillofacial surgeon and the treating orthodontist, overseeing the presurgical molding (Shetye, 2016). Further functional impairments often emerge later and a close follow-up during growth is essential for the early identification of treatment needs.

Different concepts to organize the follow-up for patients with clefts have been suggested and implemented (Hemprich et al., 2006; Capone and Sykes, 2007; Taib et al., 2015; Salimi et al., 2019). Most agree on the importance of an interdisciplinary approach with professionals from different specialties addressing their area of expertise on the one hand and on the other hand cooperating closely with the other participating specialists to achieve the optimum outcome at all levels.

The composition of the cleft core team is similar at most centers and there is a broad consensus insofar as that examinations should take place regularly (Capone and Sykes, 2007; Kasten et al., 2008; McGrattan and Ellis, 2013). The frequency of checkups is, however, not predefined and handled differently. While some centers aim at a close periodical follow-up once or twice a year during growth, others have tried to adapt the frequency of evaluations to developmental stages (Hartzell and Kilpatrick, 2014). The intensity of treatment needs seems to depend not only on the developmental stage though, but also on the cleft entity the patient was born with. For example, it could be expected that a patient with isolated cleft lip (CL) is less affected by problems of middle ear ventilation and speech forming, while a patient with isolated cleft of the soft palate (CP) usually faces less esthetical impairment and nasal obstruction.

In a previous study Lethaus et al looked into the general treatment demand of patients with cleft lip with or without palate (CL/P) during growth, identified in the clinic's annual interdisciplinary follow-up (Lethaus et al., 2021). The aim of the present study was to determine significant differences in treatment needs depending on the anatomical structures affected by cleft formation. We evaluated number and timing of treatment recommendations given to different cleft groups in the context of the regular follow-up at the tertiary care center. As a working hypothesis we assumed that patients with different cleft entities would show differing periods with intensified need of medical attention and considered the possibility to adapt the frequency of follow-up examinations accordingly.

## Patients and Methods

The follow-up consultations of the tertiary care center are offered to all patients born with a cleft malformation once a year. In case of specific problems, additional visits are scheduled. Newborn patients with CL/P are integrated into this recall program after completion of the initial reconstruction of CL, nostril, oral vestibule, and soft and hard palate, which is usually after their first year of life. Consultation is held by the core cleft team, which consists of maxillofacial surgeons, orthodontists, speech therapists, and Ear-Nose and Throat (ENT) physicians. The patient is usually seen and examined by a specialist of each of those 4 disciplines upon every visit. When required, an interdisciplinary case discussion and therapy planning takes place subsequently.

In this study, the data of all patients attending these consultation hours between June 2005 and August 2020 was analyzed retrospectively. All patients with a diagnosis of an orofacial cleft were included and gender, age, cleft entity, clinical symptoms, known syndrome, and the therapy recommendations given by the specialists were recorded. With 1126 patients included, there were 3470 appointments observed. The patients were subdivided into 17 age groups representing every year of life and summarizing patients 16 years and older. Additionally, 3 groups of cleft entities were defined according to the anatomical subunits: (1) isolated clefts of lip or lip and alveolus (CL ± A), (2) isolated CP, (3) complete clefts of lip, alveolus and palate (CLP). The analysis focused mainly on the timing, frequency and type of treatment demand testified by the participating specialists in the different cleft groups.

Statistical analyses were performed with IBM SPSS (version 27; International Business Machines Corp.) and the free software environment R. We applied Pearson's chi-squared test to sets of unpaired categorical data to evaluate the probability that observed differences were due to chance. Where sample sizes dropped below required numbers, Fisher's exact test was used. The results of the statistical hypothesis tests were deemed significant when *P* < .05. After applying a Bonferroni correction for multiple testing, the functional *P*-value threshold changed to *P* < .004. The software environment R was again used for graphical representation of data.

## Results

The study included the data of 1126 patients who showed up for a regular follow-up, resulting in a total number of 3470 consultations analyzed. The mean age of all patients was 10.2 years, 58% (*n* = 643) of patients were male, 43% (*n* = 483) were female. Patients with uni- or bilateral CLP constituted the largest group (*n* = 537), followed by patients with CP (*n* = 371). The third group included patients with uni- or bilateral CL ± A (*n* = 218). Syndromic disease was found in 3.2% of all patients. Most frequently observed were Goldenhar syndrome and DiGeorge syndrome, each found in 0.5% of all cases. Other syndromes like van der Woude, Kabuki, Franceschetti, and Down's Syndrome were less frequent in our cohort. A Pierre Robin Sequence was found in 2.8% (*n* = 32) of patients.

The percentage of therapy recommendations given throughout the entire examination period is shown in Table 1 broken down by groups. The Chi-Square test indicates a significant association between frequency of treatment recommendations and the entity of cleft (Table 1). Orthodontic treatment was recommended significantly more often to patients with CLP and less often to patients with isolated CP. Those patients on the other hand received speech therapy significantly more often compared to patients with other cleft entities. Secondary correction of the lip, rhinoplasty, and orthognathic surgery were mostly performed on patients with CLP.

**Table 1.** Overall Percentage of Patients Receiving Treatment Recommendations Throughout All Examinations and *P*-Values Resulting From Chi-Square and Fisher's Exact Test for the Association Between Cleft Entity and Treatment Recommendation.

Therapy recommended (*n* total)	CLP		CL ± A		CP	
	% (*n*)	*P*	% (*n*)	*P*	% (*n*)	*P*
Speech therapy (1261)	54.6 (293)	.340460	44.0 (96)	.000070*	65.2 (242)	.000013*
Orthodontic therapy (1202)	53.3 (286)	*1.3545* *×* 10^−9^*	38.1 (83)	.054690	33.7 (125)	.000001*
Dental treatment (272)	18.6 (100)	.729834	16.1 (35)	.359413	18.9 (70)	.686612
ENT treatment (139)	9.7 (52)	.356373	5.5 (12)	.006765	14.8 (55)	.001127*
Occupational therapy/physiotherapy (134)	8.4 (45)	.970660	5.0 (11)	.049656	10.2 (38)	.107175
Extraction/tooth exposure (119)	12.1 (65)	.006256	11.0 (24)	.428609	5.1 (19)	.000356*
Osteoplasty of the jaw (103)	16.0 (86)	*9.3512* *×* 10^−16^*	6.4 (14)	.155241	–	–
Rhinoplasty (67)	10.2 (55)	*8.2467* *×* 10^−10^*	3.7 (8)	.138201	0.5 (2)	*2.0183* *×* 10^−9^*
Lip correction (32)	4.5 (24)	.000778*	3.2 (7)	.645426	–	–
Orthognathic surgery (25)	4.3 (23)	*5.5516* *×* 10^−7^*	0.5 (1)	.065617	0 (0)	*.000085*
Velopharyngoplasty (16)	0.6 (3)	.022502	–	–	3.5 (13)	*.000090*
Implantation (15)	2.0 (11)	.065892	0.5 (1)	.327261	0.8 (3)	.409039
Correction of frenula (10)	0.4 (2)	.291522	0.9 (2)	.655971	1.1 (4)	.450682
Closure of oronasal fistula (9)	0.9 (5)	.743750	–	–	1.1 (4)	.487362

Note: Accepted level of significance after Bonferroni correction at *P* *<* *.004, s*ignificant numbers presented in italics and marked with an asterisk.

Abbreviations: CLP, clefts of lip, alveolus and palate; CL ± A, clefts of lip or lip and alveolus; CP, cleft of the soft palate.

**Figure 1.**
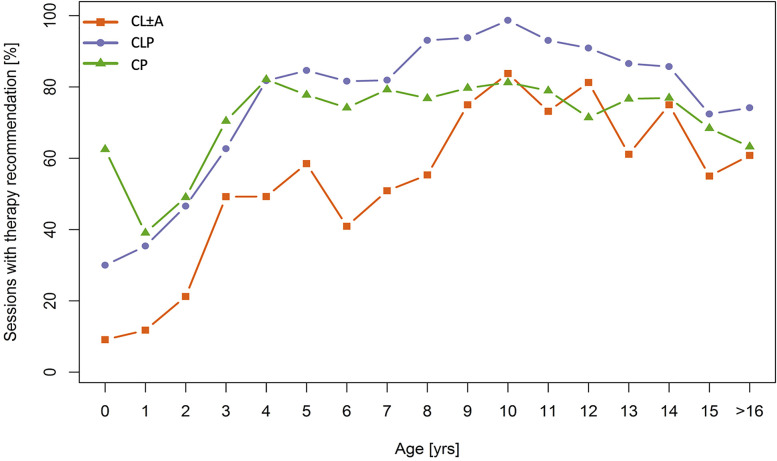
Proportion of Sessions in Which a Therapy Recommendation Was Given.

Figure 1 shows the percentage of sessions with any kind of treatment recommendation broken down by age in years. Noticeably, above the age of 2 years more than one-third of all patients received a treatment recommendation. Above the age of 3 years a treatment recommendation was given in 50% or more of consultations with only 1 outlier pertaining to patients with CL ± A at the age of 6 years. At the age of 10 years, 98% of patients with CLP and around 80% of patients with CP and CL ± A received a treatment recommendation.

**Figure 2.**
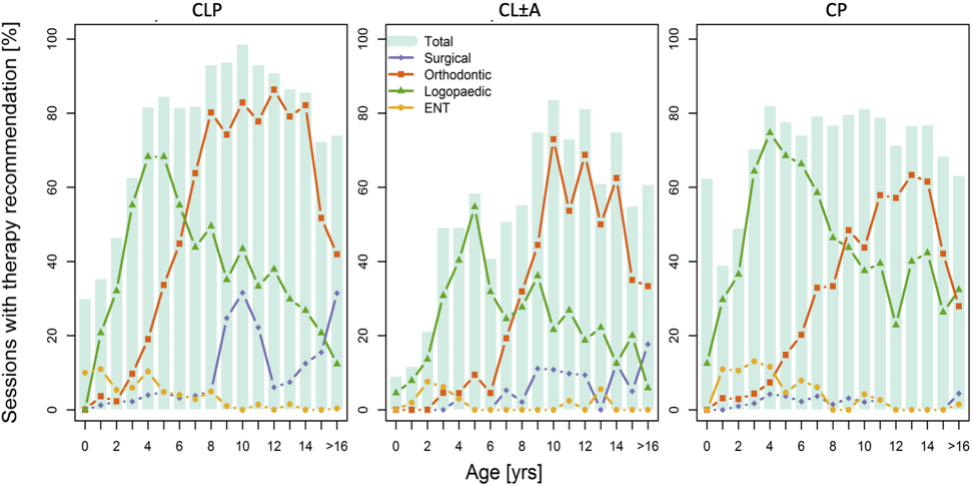
Therapy Recommendations Throughout the Age Groups for Each Cleft Entity.

In Figure 2, the frequencies of the most important therapy recommendations are compared. It is obvious that in all 3 cleft groups the onset of logopaedic therapy is indicated earlier than orthodontic treatment. In patients with isolated CP, the recommendation of logopaedic therapy was given most frequently and showed a slower decline compared to patients with other cleft entities where the peak for logopaedic treatment was in earlier childhood and decreased with age.

For patients with CLP the need for orthodontic therapy steadily increased with age between 2 and 8 years with more than 80% of patients receiving orthodontic therapy between the age of 8 and 14 years. Compared to patients with other types of clefts, this increase is more obvious, and the total percentage of orthodontic treatment is significantly higher. Surgical therapy was required mostly between age 8 and 12 for patients with clefts involving the alveolar ridge. In all 3 groups, the indication for surgical therapy goes up at the age of 16 years and above which is mainly attributable to orthognathic surgery and esthetic corrections. It is important to mention that the figure distorts the proportions in this age group since all patients above this age were summed up whereas it gives a graphically comparable representation of the other age groups.

Table 2 shows the *P*-values of associations of age and therapy recommendations resulting from Chi-square test and Fisher's exact test, respectively. Especially the association between age and orthodontic therapy and the association between age and logopaedic therapy was significant in all 3 groups.

**Table 2.** *P*-Values Resulting From Chi-Square and Fisher's Exact Test for Associations Between Age and Therapy Recommendation.

Therapy recommended (*n* total)	CLP	CL ± A	CP
Speech therapy (1261)	0.000499*	0.000499*	0.000499*
Orthodontic therapy (1202)	0.000499*	0.000499*	0.000499*
Dental treatment (272)	0.002499*	0.07446	0.009995
ENT treatment (139)	0.000499*	0.08996	0.009995
Occupational therapy/physiotherapy (134)	0.000499*	0.3818	0.02699
Extraction/tooth exposure (119)	0.07246	0.01899	0.006997
Osteoplasty of the jaw (103)	0.000499*	0.000499*	–
Rhinoplasty (67)	0.000499*	0.000499*	0.1209
Lip correction (32)	0.05247	0.1294	–
Orthognathic surgery (25)	0.000499*	0.5857	–
Implantation (15)	0.000999*	0.5822	0.0299
Velopharyngoplasty (16)	0.03198	–	0.7931
Correction of frenula (10)	0.925	0.8006	0.6712
Closure of oronasal fistula (9)	0.7176	–	0.986

Note: Accepted level of significance after Bonferroni correction at *P* < .004, significant numbers presented in italics and marked with an asterisk.

Abbreviations: CLP, clefts of lip, alveolus and palate; CL ± A, clefts of lip or lip and alveolus; CP, cleft of the soft palate.

## Discussion

The optimum treatment protocol for patients with congenital cleft malformation has been discussed for decades, also and in particular with regard to interdisciplinarity and long-term care (Meijer, 1968; Wiesinger and Hausamen, 1983; Poole, 1995; Stal et al., 1998). While the interdisciplinary composition of the cleft team has for long been a standard universally accepted, the long-term follow-up of patients with CL/P is managed differently with varying standards not only internationally but also in adjacent medical centers. The approaches to organize follow-up include: (1) individually determined intervals, arranged with the patient depending on the individual situation (Vargervik et al., 2009); (2) varying intervals depending on age and developmental status of the patient (Hartzell and Kilpatrick, 2014); and (3) regular examinations, mostly once in a year, at least up to the end of growth (Hemprich et al., 2006).

In the study cohort, patients with CL/P are examined annually after the primary surgical reconstruction is completed. Patients are asked to return in their birth month every year; as this is easy to remember it is meant to increase attendance rate and guarantee a close supervision. Analyzing the data of our follow-up program from the years 2005 to 2020 we had found that the scheduled examinations led to an important therapy recommendation in a high proportion of all 3470 consultations considered (Lethaus et al., 2021). On the other hand, we had to take notice of the fact that the majority of patients did not attend annually. Although one could suppose that families identifying a certain problem show a higher probability to attend the consultation hour, this combination of findings—constant high treatment needs throughout the years and a low attendance rate—is obviously problematic and might compromise the therapeutic outcome. Attendance rates throughout the years of follow-up have seldom been evaluated. Sparse reports indicate though this could be a common problem, arising when the effort outbalances the perceived benefits (Pfeifauf et al., 2019; Trivedi et al., 2021).

Patients with a cleft malformation and their families have recurrent and frequent contact with the health care system and a high number of different physicians and other health care professionals that possibly leads to a certain weariness over the years. Furthermore, it is a commonly known fact that patients with different cleft entities face different challenges and go through different phases of intensified treatment need. We therefore wanted to find out how and to what extent the treatment needs differed between the different manifestations of orofacial cleft malformation. Our working hypothesis was that a different phase with low treatment needs exists for every cleft group in which a lower frequency of follow-up examinations would be acceptable. Considering the results of this evaluation with the percentage of patients requiring some kind of treatment rarely dropping under 50 we had to reject this hypothesis.

We still found several relevant differences between the 3 cleft groups, especially concerning orthodontic and speech therapy as well as surgical treatment. The varying rates and timing of secondary surgical interventions are easily explained by the frequent need for secondary osteoplasty of the jaw, represented graphically by the peaks around the age of 10 years in patients with CL ± A and CLP which is not present in patients with CP (Figure 2). The need for secondary esthetic corrections and orthognathic surgery seemed to be highest in patients with CLP followed by patients with CL ± A, while we found consistently low numbers of recommended secondary surgical intervention in patients with CP.

It has been reported repeatedly that patients with CL/P present a significantly higher incidence of occlusal anomalies in primary and permanent teeth compared to the general population. The most frequent anomaly is a posterior or anterior cross bite (el-Koutby and Hafez, 1993; Vallino et al., 2008). The tension caused by the scars of primary lip and palate reconstruction restricts the sagittal maxillary growth which leads to the pathognomonic skeletal Class III while the transversal growth is as well inhibited, often resulting in a posterior crossbite (Freitas et al., 2012). Frequent dental anomalies make the orthodontic treatment even more challenging. To the best of our knowledge, there is scarce evidence on the differences of orthodontic treatment need between different cleft forms. Our data demonstrates that there is a high demand for orthodontic therapy in children and adolescents with any type of cleft. The need seems to be highest in patients with uni- or bilateral CLP. The data we gathered indicates clearly that especially the second dentition should be monitored closely to prevent missing a required start of therapy.

Furthermore, dysgnathia of varying degree is common with skeletal discrepancies in sagittal, transverse, and coronal planes. Orthognathic surgery was recommended to 4.3% of patients with CLP and 0.5% of patients with CL ± A in our cohort, but to no patient with CP. This is considerably lower than numbers found in literature, where orthognathic surgery was deemed necessary in 24% to 26% (Cohen et al., 1995), 48.3% to 65.1% (Daskalogiannakis and Mehta, 2009), 4%, 7%, 17%, 45%, and 50% (Mølsted et al., 2005) and 18.29% (Rosenstein et al., 2003), respectively. A certain bias might be due to the fact that at our clinic there is a separate consultation hour held for patients with dysgnathia, so that a significant number of patients might have been referred there directly and were not registered in our follow-up.

The numbers of ENT treatment recommendation are highest during the first years of life, but are generally low in our study, which might partly result from our study design. We evaluated all doctor’s letters from our interdisciplinary consultation hours. In some cases, the ENT findings and recommendations were not integrated because of separate documentation.

The indication for speech therapy follows a similar curve in all 3 cleft groups with a peak during the years immediately preceding school enrollment. While the high need for speech therapy in patients with clefting of the hard and soft palate is in accordance with numbers found in literature (Sell et al., 2001; Rezaei et al., 2020), the only slightly lower numbers in patients without affection of the palate is surprising. In 2021, Smarius et al published their results of a prospective study evaluating the need for speech therapy in a cohort of patients with CL only, finding that in 24% of patients speech therapy was indicated and concluding that there is no higher risk of speech problems compared to the general population (Smarius et al., 2021). However, there was only a single assessment of speech and language at the age of 3 years. At that age, speech therapy was recommended to 30% of our patients, while at the age of 5 years, speech pathology was found in 54% of our patients resulting in the recommendation of speech therapy. Discrepancies might be partly due to the inclusion of patients with alveolar clefting in our group. In a different cohort of 92 patients with CL ± A—with alveolar involvement in half of the cases—the indication of speech therapy was confirmed in 23% of patients, while this was in no case attributed to lip abnormalities (Padovano et al., 2020).

Data on follow-up and outcome of patients with isolated CL is limited as they are rarely evaluated as a distinct group. In compliance with our initial hypothesis that patients without palate involvement might need less intense follow-up, the recently published study mentioned above comes to the conclusion that there is no need for a constant follow-up of patients with CL only (Padovano et al., 2020). However, there are others warning against denying these children continuous team care as cleft-associated abnormalities are frequent and a late diagnosis could worsen the outcome (Vallino et al., 2008). Judging from our data, a regular multidisciplinary assessment of patients with CL cannot be dismissed.

With regard to the high drop-out rate, the results of our study demand us to think about ways to increase patients’ and parents’ motivation to regularly attend the interdisciplinary consultation hours as this may have a decisive effect on the treatment outcome. One evident idea would be to contact all patients by phone in their birth month and schedule an appointment directly. The implementation of a booklet including information, medical records, and a participation card similar to the one used for pediatric checkup could support parents in keeping track of follow-up consultations. Furthermore, to develop a mobile app integrating an annual reminder to schedule a checkup appointment would be an enticing project.

## Conclusion

Although we found significant differences between the treatment needs of patients with different cleft entities, the general treatment need is high throughout the growth period. Patients with CL ± A showed a similarly high treatment demand for cleft-associated problems and should be monitored closely.

Thus, it does not seem possible to sensibly adapt the frequency and timing of follow-up consultations to the cleft entity. A close follow-up for patients with diagnosis of CL/P is crucial throughout the development and measures should be taken to increase participation in regular follow-up appointments.
